# Lay beliefs of TB and TB/HIV co-infection in Addis Ababa, Ethiopia: a qualitative study

**DOI:** 10.1186/1756-0500-4-277

**Published:** 2011-08-03

**Authors:** Mekdes K Gebremariam, Gunnar A Bjune, Jan C Frich

**Affiliations:** 1 Section for International Health, Institute of Health and Society, University of Oslo, PO Box 1130, Blindern, NO-0318 Oslo, Norway; 2 Department of Health Management and Health Economics, Institute of Health and Society, University of Oslo, PO Box 1089, NO-0317 Oslo, Norway

## Abstract

**Background:**

Knowledge about lay beliefs of etiology, transmission and treatment of TB, and lay perceptions of the relationship between TB and HIV is important for understanding patients' health seeking behavior and adherence to treatment. We conducted a study to explore lay beliefs about TB and TB/HIV co-infection in Addis Ababa, Ethiopia.

**Findings:**

We conducted a qualitative study using in-depth interviews with 15 TB/HIV co-infected patients and 9 health professionals and focus group discussions with 14 co-infected patients in Addis-Ababa, Ethiopia. We found that a predominant lay belief was that TB was caused by exposure to cold. Excessive sun exposure, exposure to mud, smoking, alcohol, khat and inadequate food intake were also reported as causes for TB. Such beliefs initially led to self-treatment. The majority of patients were aware of an association between TB and HIV. Some reported that TB could transform into HIV, while others said that the body could be weakened by HIV and become more susceptible to illnesses such as TB. Some patients classified TB as either HIV-related or non-HIV-related, and weight loss was a hallmark for HIV-related TB. The majority of patients believed that people in the community knew that there was an association between TB and HIV, and some feared that this would predispose them to HIV-related stigma.

**Conclusion:**

There is a need for culturally sensitive information and educational efforts to address misperceptions about TB and HIV. Health professionals should provide information about causes and treatment of TB and HIV to co-infected patients.

## Background

The incidence of TB in Ethiopia is estimated to be 379 per 100 000 populations for all cases and the prevalence 643 per 100 000 populations [1]. According to data from the Ministry of Health, TB is the leading cause of morbidity, the third cause of hospital admission and the second cause of death in Ethiopia [2]. Ethiopia is also one of the countries worst affected by the HIV epidemic, with a total of 1.2 million people living with HIV in 2007 [3]. The TB/HIV co-infection rate is 40% [2,4].

Studies in Ethiopia found that lay misperceptions exist regarding the origin and transmission of TB, and perceived and enacted stigma related to TB has also been reported [5-8]. Misperceptions about HIV and stigmatization against patients with HIV have been found in previous research [8-10]. In addition, there is a widespread belief of an association between the two illnesses [7,11], which may predispose TB patients to HIV-related enacted and felt stigma [12]. No study in Ethiopia has specifically explored lay beliefs about the association between TB and HIV. Knowledge of lay beliefs about etiology and the association between TB and HIV may have implications for clinical management and for public health information campaigns [13]. The aim of this study is to explore lay beliefs about TB and TB/HIV co-infection in Addis Ababa, Ethiopia.

## Methods

### Participants

Participants were recruited from three health centers in Addis-Ababa, Ethiopia, in 2008. These health centers (Bole Health Center, Arada Health Center and Woreda 7 Health Center) all practice free directly observed therapy short course (DOTS) for TB and offer free anti-retroviral therapy (ART) for HIV. The centers were selected among a total of 21 health centers owned by the city administration. The three health centers are located in different parts of the city, and they were representative in terms of service provision, staffing and cultural diversity.

Data was collected using in-depth interviews and focus group discussions. In order to capture a diversity of views, we used a maximum variation sampling strategy when recruiting participants. We included participants with diversity with regards to socio-demographic background including age (mean: 36.8 years, range: 20-47), gender, education, and occupation.

Individual interviews were conducted with 15 patients (seven men, eight women) who had been on concomitant treatment for TB and HIV. Among these patients, six had completed their TB treatment, six had discontinued their TB treatment, and three were on retreatment for TB. Three were illiterate, three could read and write, four had education at primary school level, and five had education at secondary school level and above. Four of these patients were unemployed, three were formally employed, and eight had informal employment. We subsequently conducted two focus group discussions with a total of 14 patients (seven men and seven women with diverse socio-economic backgrounds) who underwent concomitant treatment for TB and HIV when the study was conducted, in order to further explore and validate our preliminary findings from the in-depth interviews.

Nine health professionals (six clinical nurses, two health officers and one doctor aged 30-55) were interviewed individually. The majority of these health professionals had over ten years of work experience. Patients were invited to participate in the study through an invitation letter provided by health personnel at the TB and at the ART clinics.

### Data collection

Audio-recorded individual interviews and focus group discussions were conducted by MK in Amharic. Individual interviews took place in private rooms, and a semi-structured format with open-ended questions was used. The questions covered participants' perceptions about the etiology and transmission of TB, and the association between TB and HIV. Some questions regarding perceptions towards HIV were also asked. Similar topics were covered in the focus group discussions, and issues that arose in the interviews were brought up for further discussion. We used this triangulation of data sources in order to increase the validity of findings in our study. Data collection was continued until the point where we ascertained that new interviews were not yielding new results, with saturation level reached.

### Data analysis

Audio-recorded data from interviews with individual patients was transcribed verbatim and translated into English. A preliminary analysis of this data was done, where lay beliefs about causes of TB and beliefs about associations between TB and HIV were identified. The patterns that were identified were subsequently explored using focus group discussions. Data from focus group discussions and health worker interviews which were also audio-recorded were also transcribed verbatim and translated into English. The final analysis involved identification and coding of material about participants' perceptions/beliefs about TB and TB/HIV using Giorgi's phenomenological method, as modified by Malterud [14]. The material was read to get an overview and subsequently units of meaning that represent different aspects of participants' beliefs/perceptions about TB and TB/HIV were identified. A list of codes was then created and the material was coded. The content of each code was condensed and summarized in order to make generalized descriptions concerning existing perceptions about TB and TB/HIV.

### Ethics

Informed consent was obtained from all participants prior to data collection. Anonymity and confidentiality were ensured. The study has been approved by the Regional Committees for Medical and Health Research Ethics (REK) in Norway and was granted ethical clearance by Addis Ababa Health Bureau in Ethiopia.

## Results

### Lay beliefs about the cause of TB

The most common factor patients thought caused TB was *bird*, which literally means cold. Many patients recalled being "hit" by *bird *some time before they started coughing. A 36 years old male participant with secondary education, re-started on anti-TB after relapse, explained:

"I have been in Italy, and there is a lot of snow there, and that is what predisposed me to TB. It is very cold there, and we wear a lot of clothes, but sometimes, mainly when you are working, you forget to wear good clothes, and the bird hits you. That is why I had TB. Bird had gone into my body."

All health professionals were aware of this belief and some referred to it as "the *bird *theory." In their experience, many patients associate cough and different chest conditions such as pneumonia and TB to exposure to *bird*. A clinical nurse shared his experience at the TB clinic:

"Over 90% of people, including educated people, are saying it is bird, and then they say I started coughing, and it changed into TB. Even educated people say that. When we tell them, cover your mouth, they get confused. And they say, but we have been closing all windows, so then should our brother, our mother, get tested? And it is even a problem here where we work. They go and close the door, and we explain, we leave it open because of this reason."

Other causes of TB patients mentioned were excessive exposure to sun, exposure to mud, smoking, alcohol, khat and inadequate food intake. Some patients had beliefs that smoking, alcohol, and khat could cause TB, and these beliefs were reinforced by health professionals' prohibition of intake of these substances upon diagnosis of TB and initiation of treatment. These substances were also believed to lead to a delay in getting cured once the patient started treatment. Lack of food or consumption of poor quality food was also thought to lead to TB by many. A 46 years old male participant who can write and read but has no further formal education and had completed his TB treatment, said:

"Q. Do you know what causes TB?

Bird, sun, excess mud. That is what predisposed me to TB. In addition, you know, there is the problem of food. If you do not eat good food, you get TB. And for me, my life is from hand to mouth, so, sometimes, you have to eat beans and sleep."

Few patients mentioned bacteria as a cause of TB. Some patients mentioned transmission from another person through breathing. Sharing eating and drinking objects was also reported by some as a mode of transmission of TB, and patients or their families had separated their eating utensils after the diagnosis of TB. Two patients said that TB could be sexually transmitted since health professionals were telling them not to have sex during treatment. Health professionals who were asked why they were prohibiting sex during TB treatment said that it was because of the fear that patients would lose too much energy. A clinical nurse explained:

"Yes, we tell them to stop having sex. You know, you lose energy with sex, and for a sick person, it is not good. If you don't tell them, they just do it without limits; you have to be strict with that."

Few patients also believed that different origins and modes of transmission exist for TB, some getting it from *bird*, others from other people, that being the reason why some patients are told to cover their mouth at the clinic, and others not, and said that the drugs were also different depending on which type of TB one has. In contrast to the knowledge about TB, all of the patients in the study knew that HIV could be transmitted sexually and through blood containing sharp objects, such as needles. Most had gotten the information from mass media, such as radio and TV.

### Associations between TB and HIV

A large majority of patients was aware of an association between TB and HIV. One view was that TB could change into HIV, if TB had been left untreated for long. A 50 year old illiterate female participant who had completed her TB treatment explained how TB changed into HIV in her case:

"It is from TB that I got this disease (HIV). They go side to side. The samba (TB) went into HIV. Initially, I was saying, can it be from sharp objects, but it is not. It is the samba that changed. I am a menekussie (equivalent to a nun in the orthodox religion); I did not go to a man."

Most health professionals were aware of this belief among patients, as noted by a clinical nurse:

"When I was doing PICT, I had patients who thought that you would have HIV if you have TB, they thought of the two diseases as being similar. At the beginning, when you tell them that a person with HIV would get TB because of the immunity..., they say, Ah! It's like that? Because they thought HIV follows TB, that they will get it after the TB. There is a patient who recently told me that the TB changed on him."

For the majority of these patients, two types of TB existed: TB related to HIV (or that could change into HIV), and the one that was not related to HIV. The most common type of TB was believed to be HIV-related TB. Other patients explained the relationship between the two illnesses as being a result of a weakness in the body due to HIV, which leads to a susceptibility to other diseases such as TB, but also diarrhea and skin lesions.

Health professionals reported that patients who came to the TB clinic, and the family members who accompanied them, usually assumed the condition was caused by HIV infection.

Patients tended to classify other patients with TB as having HIV or not based on their weight status and their strength. Particular emphasis was put on physical appearance, with thin patients classified as being HIV positive; weakness and darkening of the skin were also believed to be other signs of HIV. A 46 years old male participant with completed secondary education, who had defaulted TB treatment explained:

*"When I see other patients in the TB clinic who do not have HIV, they are stronger than me*.

Q. How do you know they are HIV negative?

I told you, they are strong. They are not like me. The two illnesses, they support each other to weaken you. Oh! Your body, your mind, they all get weak!"

Some patients believed the association between TB and HIV was known in the community, and they reported not disclosing their diagnosis of TB in fear of being suspected for having HIV and experiencing stigmatization. Although both TB and HIV were considered as stigmatized conditions, patients reported that there was more stigma attached to HIV. Some patients said that the media was responsible for the fact that people were automatically associating TB and HIV, an opinion shared by health professionals. A clinical nurse explained:

"You know, the people they interview on the media usually say: I had TB and I was told I am (HIV) positive. So for those who listen to it that would mean: ok, if there is TB, there is HIV."

## Discussion

Our study documented different lay beliefs with regards to TB and TB/HIV co-infection among patients in Addis Ababa, Ethiopia. Implications of these beliefs are discussed below.

### Beliefs about etiology and transmission

Misperceptions about etiology and transmission of TB have been previously reported [5-7,15,16]. The widespread belief in *bird *(cold) as a causative agent for TB is significant for two reasons. First, it may make patients revert to self-treatment, delaying visit to health facilities, as has also been indicated in a previous qualitative study from Ethiopia [7]. Second, this causal belief could be an explanatory factor for the patients' delay in attending TB treatment [17-19]. This delay is particularly problematic for patients co-infected with HIV, since an untreated TB infection causes further deterioration in the patient's immune status [20]. Patients come at a late stage for treatment when their symptoms have progressed, which makes clinic attendances difficult, tolerability of drugs worse, worsen the economic situation of both the patient and/or families because of inability to work which in turn might adversely influence treatment. The belief in *bird *as a causal mechanism needs to taken into account in TB prevention efforts [4].

### Beliefs about the association between TB and HIV

The view that TB transforms into HIV, expressed among our participants, has previously been found in a study from Addis-Ababa [7]. Some patients considered TB as a sign of HIV infection and our study indicates that this belief might also exist among TB patients without HIV. A recent study from Ethiopia similarly found that patients with TB/HIV had a high degree of perceived stigma as compared to patients with TB only [21], and our results explain, at least in part, the difference in perceived stigma in the two patient groups.

Our results suggest that while patients' knowledge about the cause of TB was poor they reported adequate knowledge about the major routes of transmission of HIV. This finding is in concordance with a study from Thailand which found that the community had a higher awareness about HIV as compared to TB [22]. This might be a result of the coverage of HIV in the media and education campaigns, which has not been the case for TB.

### Methodological considerations

This study is based on interviews with a total of 29 patients and 9 health professionals and is limited to an urban setting. Our findings reflect a diversity in beliefs; and we triangulated data sources in order to strengthen the validity of our results. We thus believe our study can provide valuable insights into lay perceptions of TB and TB/HIV, and that our findings could inform quantitative studies of lay beliefs of TB and TB/HIV co-infection.

## Conclusion

There is a need for culturally sensitive information and educational efforts to address misperceptions about TB and HIV. Health professional should provide information about causes and treatment of TB and HIV to co-infected patients.

## Competing interests

The authors declare that they have no competing interests.

## Authors' contributions

MK designed the study in collaboration with GAB and JCF. MK collected the data. MK and JCF developed a list of codes. MK coded the data and all authors contributed in the subsequent analysis. All authors have critically revised the manuscript.
